# Supplementary material for: Identification of Chromoblastomycosis and Phaeohyphomycosis Agents through ITS-RFLP
Source: J Fungi (Basel). 2024 Feb 18;10(2):159. doi: 10.3390/jof10020159 (PMC10890301; doi:10.3390/jof10020159)
Supplement: Supplementary file 1 [file jof-10-00159-s001.zip › Figure S1.pdf]

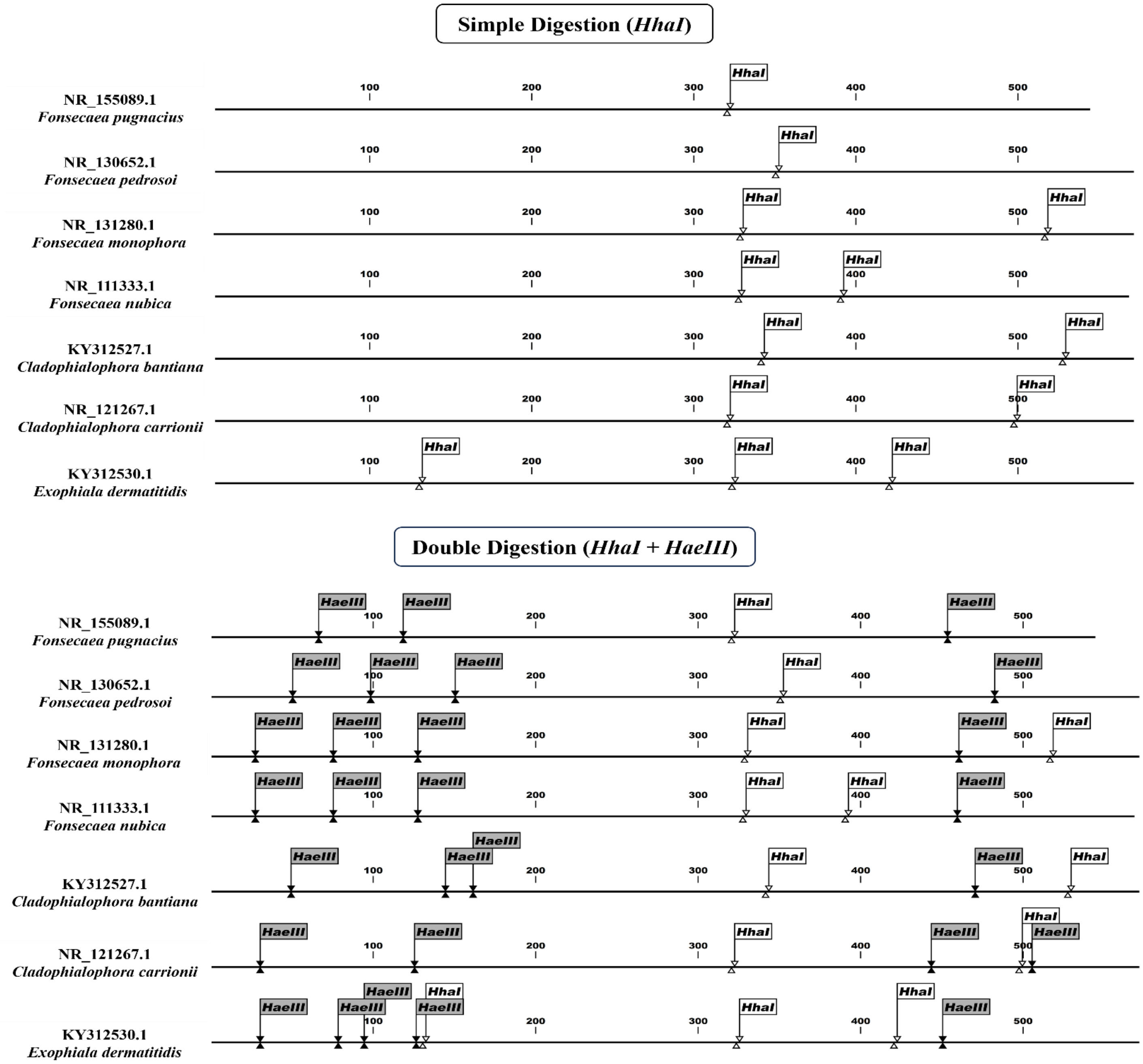


**Figure S1**: In silico analysis with sequences from less prevalent agents of chromoblastomycosis.
