# Supplementary material for: Identification of Chromoblastomycosis and Phaeohyphomycosis Agents through ITS-RFLP
Source: J Fungi (Basel). 2024 Feb 18;10(2):159. doi: 10.3390/jof10020159 (PMC10890301; doi:10.3390/jof10020159)
Supplement: Supplementary file 1 [file jof-10-00159-s001.zip › Table S1.pdf]

Table S1: Sequences used in the in silico analyses and Genbank code access.

Genbank Access	Species
MF416923.1	*Fonsecaea pedrosoi*
MF416922.1	*Fonsecaea pedrosoi*
MF416919.1	*Fonsecaea pedrosoi*
KY312537.1	*Fonsecaea pedrosoi*
KY312536.1	*Fonsecaea pedrosoi*
KY312535.1	*Fonsecaea pedrosoi*
KY312533.1	*Fonsecaea pedrosoi*
KY312532.1	*Fonsecaea pedrosoi*
KY312531.1	*Fonsecaea pedrosoi*
KY312529.1	*Fonsecaea pedrosoi*
KY312528.1	*Fonsecaea pedrosoi*
KY312526.1	*Fonsecaea pedrosoi*
KY312525.1	*Fonsecaea pedrosoi*
KY312524.1	*Fonsecaea pedrosoi*
KY312523.1	*Fonsecaea pedrosoi*
NR_130652.1	*Fonsecaea pedrosoi*
KP132210.1	*Fonsecaea pedrosoi*
KP132209.1	*Fonsecaea pedrosoi*
KP132205.1	*Fonsecaea pedrosoi*
KC709978.1	*Fonsecaea pedrosoi*
MF416924.1	*Fonsecaea monophora*
MF416920.1	*Fonsecaea monophora*
KY312538.1	*Fonsecaea monophora*
KY312534.1	*Fonsecaea monophora*
NR_131280.1	*Fonsecaea monophora*
MH382070.1	*Fonsecaea monophora*
MH382069.1	*Fonsecaea monophora*
MH382068.1	*Fonsecaea monophora*
MH382067.1	*Fonsecaea monophora*
MH382066.1	*Fonsecaea monophora*
MH382065.1	*Fonsecaea monophora*
KP132197.1	*Fonsecaea monophora*
KP132193.1	*Fonsecaea monophora*
KC614700.1	*Fonsecaea monophora*
EU285272.1	*Fonsecaea monophora*
KY312527.1	*Cladophialophora bantiana*
KY432483.1	*Cladophialophora bantiana*
KP131826.1	*Cladophialophora bantiana*
KP131825.1	*Cladophialophora bantiana*
KP131824.1	*Cladophialophora bantiana*
AY366924.1	*Cladophialophora bantiana*
AF131079.1	*Cladophialophora bantiana*
MH094277.1	*Cladophialophora bantiana*
KY312530.1	*Exophiala dermatitidis*

NR_121268.1	*Exophiala dermatitidis*
KP132044.1	*Exophiala dermatitidis*
KP132043.1	*Exophiala dermatitidis*
KP132042.1	*Exophiala dermatitidis*
KP132041.1	*Exophiala dermatitidis*
KP132040.1	*Exophiala dermatitidis*
NR_121267.1	*Cladophialophora carrionii*
MK820048.1	*Cladophialophora carrionii*
MK820047.1	*Cladophialophora carrionii*
MK820046.1	*Cladophialophora carrionii*
NR_155089.1	*Fonsecaea pugnacius*
MH444809.1	*Fonsecaea pugnacius*
KR706554.1	*Fonsecaea pugnacius*
NR_111333.1	*Fonsecaea nubica*
KY432481.1	*Fonsecaea nubica*
AY366931.1	*Fonsecaea nubica*
